# Combined treatment with Rg1 and adipose-derived stem cells alleviates DSS-induced colitis in a mouse model

**DOI:** 10.1186/s13287-022-02940-x

**Published:** 2022-06-21

**Authors:** Rui Zhang, Qingqing Zhang, Yanni Chen, Qing Zhao, Bo Zhang, Ling Wang, Chungen Zhou, Qi Zhang, Kun Chen, Yuqing Zhang, Xiaotao Hou, Hao Chen, Xingyin Liu, Min Ni, Bin Jiang

**Affiliations:** 1 Anorectal Surgery of Zhongda Hospital Southeast University, Nanjing, 210009 China; 2 Department of Pathogen Biology, Key Laboratory of Pathogen of Jiangsu Province, Nanjing Medical University, Nanjing, 211166 China; 3 Graduate School of Nanjing University of Chinese Medicine, Nanjing, 210029 China; 4 Colorectal Disease Center of Nanjing Hospital of Chinese Medicine, Affiliated to Nanjing University of Chinese Medicine, Nanjing, 210022 China

**Keywords:** Ginsenoside (Rg1), Adipose-derived stem cell (ADSC), Colitis, Gut microbiome

## Abstract

**Background:**

Inflammatory bowel diseases, consisting of Crohn’s disease and ulcerative colitis constitute chronic inflammatory conditions that may compromise the whole gastrointestinal tract as well as the colonic mucosa. Currently, there are no curative interventions for IBD, and all available treatments have side effects that limit their use. Adipose-derived stem cell (ADSC) treatment is a prospective treatment option for IBD. Previous findings indicated that ginsenoside (Rg1) dampened inflammatory diseases like colitis by inhibiting the binding of LPS to TLR4 on macrophages and restoring the Th17/Treg ratio. The purpose of this work was to investigate whether Rg1 can increase the influence of ADSC in a mouse model of colitis triggered by dextran sulfate sodium (DSS).

**Methods:**

ADSC was intravenously inoculated into mice with DSS-triggered colitis, while Rg1 was delivered via oral gavage. Colon inflammation was assessed via body weight, colon length along with H&E staining. Serum cytokine levels were measured using ELISA. Besides, flow cytometry was adopted to determine the percentage, as well as FMI of immune cells in the spleen. The effects of simultaneous Rg1 and ADSC treatment on TLR4-MyD88 signaling were assessed via immunofluorescence.

**Results:**

Rg1 and ADSC effectively alleviated the impacts of colon inflammation, weight loss, and colon length reduction along with histological score. Treatment with Rg1 and ADSC reduced serum levels of the proinflammatory cytokines, IL-1β, TNF-α, IL-6, IL-4, and IL-17A and upregulated the level of immunosuppressive cytokine, IL-10. Compared with ADSC or Rg1 alone, combined treatment with Rg1 and ADSC significantly improved the structure of microbial community. Additionally, treatment with Rg1 plus ADSC selectively elevated the level of splenic regulatory T (Treg) cells and downregulated the proportion of T helper type 17 (Th17) cells, indicating restoration of intestinal homeostasis. Besides, we established that the combination of ADSC + Rg1 restored immunological balance more effectively than either ADSC or Rg1 alone, illustrating that Rg1's modulatory function on the gut microbiota may boost the impact of ADSCs in restoration of the immune balance. ADSC combined with Rg1 might downregulate the expression of TLR4 and MyD88, thereby suppressing TLR4-MyD8 signaling. The immunofluorescence results also suggested that co-therapy with Rg-1 and ADSC may optimize treatment strategies of IBD.

**Conclusions:**

Here, we find that the combination of Rg1 and ADSC alleviates DSS-induced colitis in a mouse model more efficiently than ADSC alone, indicating that Rg1 enhances the effect of ADSC against colitis.

**Supplementary Information:**

The online version contains supplementary material available at 10.1186/s13287-022-02940-x.

## Background

Crohn’s disease (CD) and ulcerative colitis (UC), the two clinically defined forms of inflammatory bowel disease (IBD), are chronic remittent or progressive inflammatory conditions that can affect the entire gastrointestinal tract and colonic mucosa [1]. Mounting evidences have demonstrated that environmental factors modify the interaction of gut microbiota and mucosal immune system to influence IBD development [2]. IBD is associated with substantial morbidity, poor quality of life, and colitis-associated colorectal cancer (CAC). Now, IBD has emerged as a public health challenge worldwide [3]. However, patients with IBD face debilitating treatment for life and most of the treatments for IBD are suboptimal. Therefore, to improve the prognosis of patients with IBD, more effective treatment with fewer side effects is urgently needed.

Mesenchymal stem cells (MSCs) have emerged as a potential therapeutic avenue for IBD capable of restoring epithelial barrier integrity, homing to the damaged tissue, inhibiting inflammatory responses, and regulating immunity [4–6] [7–9]. Moreover, numerous studies indicate that systemic administration of MSC alleviates colitis in a mouse model [10–13]. We and other groups have previously reported that ADSCs, a class of MSCs, are a feasible and effective treatment for Crohn’s fistula-in-ano. When compared with traditional incision and thread-drawing, ADSC therapy was well tolerated and could protect anal function, relieve pain, and allow quick recovery, thereby improving the quality of life during the perioperative period [14].

Ginsenoside Rg1 is a natural stem extract and a major active ingredient in ginseng [15, 16]. Ginsenoside Rg1 is reported to promote stem cell orientation transformation and proliferation [17, 18]. For example, Rg1 has been shown to enhance the proliferation, differentiation, and soft tissue regeneration of human breast adipose ADSCs in collagen type I sponge scaffolds in vitro and in vivo, resulting in a broad new organizational network [19]. Moreover, Zhu et al. reported that Rg1 markedly reduces the level of proinflammatory cytokines released by dendritic cells in a DSS-induced mouse model of colitis [20]. Here, we examined the effects of combined treatment with Rg1 and ADSC on a mouse model of DSS-induced colitis and investigated the underlying mechanisms.

## Materials and methods

### Isolation of adipose-derived stem cells

Human abdominal or buttock adipose tissues were collected from patients receiving regenerative medicine using ADSC at Nanjing Hospital of Chinese Medicine, Nanjing University of Chinese Medicine (Nanjing, China). Adipose tissues were collected from healthy adult male participants at Nanjing Hospital of Chinese Medicine, Nanjing University of Chinese Medicine (Nanjing, China). All study participants gave written informed consent. ADSCs were isolated from the samples and stromal vascular fractions cultured in serum-free culture media, at 37 °C, 5% CO_2_. Confluent, adherent cells were trypsinized and passaged using serum-free culture media three times, and the features of the ADSCs were verified using differentiation, proliferation, and immunologic assays. The cells were then frozen and delivered to Nanjing Medical University. After thawing, cells were immediately washed, counted, then suspended in PBS. For flow cytometry, ADSCs were harvested, washed, and incubated with antibodies against CD90-FITC, CD44-PE, CD105-FITC, CD73-PE, CD34-PE, and CD45-FITC.

### Animals

Six-to-eight-week-old male C57BL/6 mice were purchased from the experiment center of Nanjing Medical University (Nanjing, China) and housed in temperature-, humidity-, and light cycle-controlled conditions. All animal protocols complied with animal care regulations and were approved by Nanjing Medical University’s institutional animal care and use committee.

### Induction of experimental colitis and study design

Colitis was induced using 3% DSS (molecular weight 36,000–50,000, MP Biomedicals, Santa Ana, CA) in drinking water for 7 days followed by regular water for 7 days. Mice were divided into five groups (n = 10–15/group). The ADSC and ADSC + Rg1 groups were intravenously injected with 1 × 10^6^ ADSCs [21] on the 4th and 7th days, while the control, Rg1, and DSS groups were injected with PBS. The Rg1 and ADSC + Rg1 groups were also administered Rg1 (20 mg/kg) [24] by oral gavage, once daily from the 1st to 14th day (starting with DSS treatment), while other groups received PBS. All mice were killed on the 15th day after the start of the experiment.

### Body weight and colon length assessment

To evaluate the therapeutic effects of ADSCs and Rg1, body weight and colon length were monitored. Body weight was recorded daily (n = 10–15/group). Colon length was measured from the anus to the cecum immediately after harvesting the colon (n = 10–15/group). Samples were measured as an indirect assessment of inflammation.

### Histopathological analysis

Histological score was calculated as follows (n = 6/group). Colon samples were fixed in 10% formalin, paraffin-embedded, and sectioned at 4 µm. After H&E staining, histological evaluation was done in a blinded manner using a previously published scoring system [22] as follows: (a) a score of 1 indicates mild mucosal inflammatory cell infiltration into intact epithelium; (b) a score of 2 indicates inflammatory cell infiltration into mucosa and submucosa with undamaged epithelium; (c) a score of 3 indicates mucosal infiltration with focal ulceration; (d) a score of 4 indicates mucosal and submucosal infiltration by inflammatory cells and focal ulceration; (e) a score of 5 indicates moderate mucosal and submucosal infiltration by inflammatory cells with extensive ulceration; and (f) a score of 6 indicates transmural inflammation and extensive ulceration.

### Analysis of serum parameters

Blood was collected from all mice and sera analyzed for the levels of the cytokines, IL-10, IL-6, IL-17A, IL-33, IL-1β, and TNF-α (n = 8/group) following kit manufacturer instructions. All assay kits were purchased from R&D System.

### Flow cytometry

Spleens were harvested and mechanically dissociated into single cell suspensions. The splenocytes were then incubated with Fc block (CD16/32) for 10 min to block non-specific binding and stained with the conjugated antibodies. For surface staining, cells were incubated with specific antibodies for 30 min at 4 °C and washed twice. For intracellular cytokine staining, cells were stimulated with phorbol myristate acetate and ionomycin, in the presence of monensin (eBioscience), for 5 h before incubation with BD fixation/permeabilization solution. Treg cells were fixed and permeabilized using eBioscience Foxp3/transcription factor staining buffer kit (Invitrogen) according to manufacturer instructions. Flow cytometry was then done on a BD FACSVerse flow cytometer and data analyzed using FlowJo version 10.

### DNA isolation and 16S rRNA gene sequencing

Bacteria genomic DNA was extracted from 100 mg of stool sample using a DNA extraction kit (#DP328, Tiangen Company, Beijing, China) following manufacturer instructions. DNA concentration and purity were determined on a Qubit 2.0 Fluorometer (Thermo Scientific, USA) and the V3 and V4 16S rRNA regions amplified using composite specific primers (338F: 5’-ACTCCTACGGGAGGCAGCAG-3’ and 806R: 5’-GGACTACHVGGGTWTCTAAT-3’). The 16S rDNA data were analyzed using QIIME software package 2.0. All analyses and calculations were done using in-house Perl scripts as described previously [23, 24].

### Immunofluorescence

Immunofluorescence was used to determine the levels of TLR4 and MyD88. All sections incubations were performed at room temperature and rinsed with PBS after incubation. The sections were deparaffinized in xylene and rehydrated in graded alcohol. They were then blocked with 10% normal goat serum for 30 min at room temperature. The sections were then incubated overnight with primary antibodies against TLR4 and MyD88 (1:200 dilution, Affinity Biosciences, Cincinnati, USA) at 4 °C. They were then washed with PBS and incubated with Cy3-conjugated goat anti-rabbit secondary antibodies (1:300 dilution, Servicebio, Wuhan) at 37 °C for half an hour in the dark. They were then washed with PBS, counterstained with DAPI for 10 min, and mounted.

### Statistical analysis

Unless otherwise indicated, data are presented as mean ± SD, and all data analyses were done on GraphPad Prism 8.0 (GraphPad Software, Inc.). Correlation tests were done using Spearman’s rank correlation analysis. The alpha diversity index was analyzed using QIIME (Version 2.0). PCoA was done using the ade4 package on R (Version 3.4.4). Two-tailed * P* ≤ 0.05 was signified statistical significance.

## Results

### Characterization of human ADSCs

ADSCs expressed all specific MSC markers (CD73, CD105, CD90, and CD44) but not the hematopoietic markers (CD34 and CD45). The ADSCs differentiated into adipocytes, chondrocytes, and osteocytes (Additional file 1: Fig. S1).

### ADSC and Rg1 administration ameliorated DSS-induced colitis

Monitoring of body weight showed that mice in the DSS group consistently lost weight from the 5th day. However, combined ADSC and Rg1 treatment had a much better weight regain compared with ADSC and Rg1 treatment alone (P ≤ 0.05). In the colitis model, disease severity correlates with colon length shortening due to intestinal inflammation [25]. Colon length analysis revealed significantly shorter colons in the DSS group when compared with the ADSC, Rg1, and ADSC + Rg1 groups (P ≤ 0.05).

**Fig. 1:**
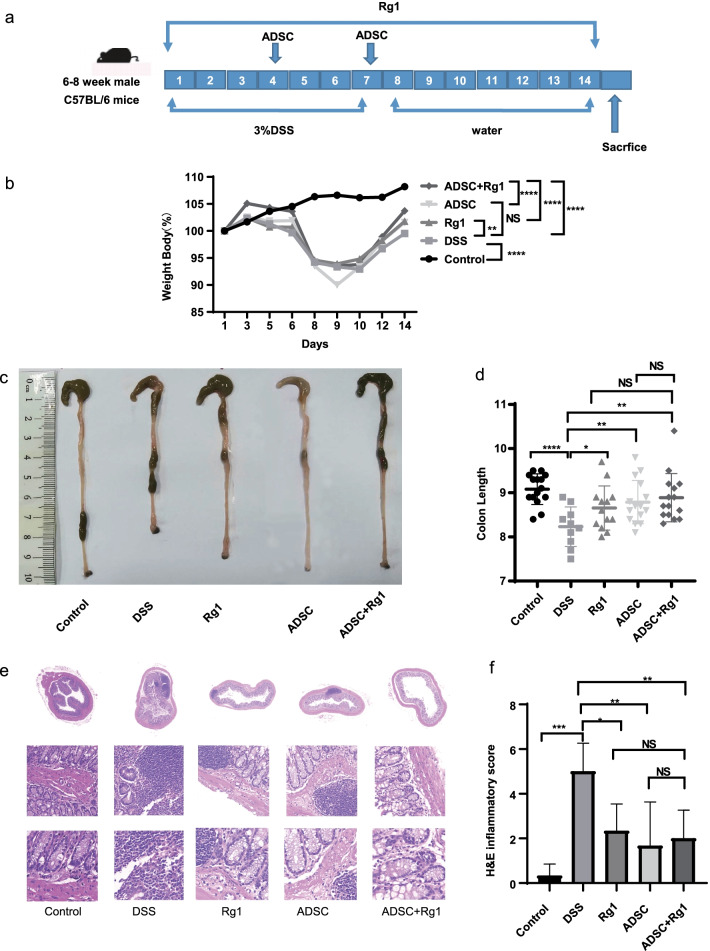
Mice administrated by ADSC, Rg1, or ADSC + Rg1 are more tolerance to DSS-induced colitis. **a** Scheme of the animal experimental design. Mice were assigned to five groups randomly (control groups; DSS groups; Rg1 groups; ADSC groups; and ADSC + Rg1 groups). Mice were administered 3% DSS for 7 days followed by normal drinking water. At day 4 and 7 of DSS treatment, mice in ADSC and ADSC + Rg1 groups were intravenously injected with ADSC. Mice in Rg1 and ADSC + Rg1 groups were administered with Rg1 daily by oral gavage. On day 15, mice were killed. **b** Body weight change and final survival at day 15. n = 10–15. **c** Image displays the gross colon appearance. **d** Colon length was determined during necropsy. n = 10–15. **e** Representative H&E stained colon cross sections of mice at day 15 of the acute DSS colitis protocol. Bars, 200 μm. **f** H&E inflammatory score of colon sections. n = 6, * P < 0.05, **P < *0.01*, ns, not significant

In agreement with the previous findings [10], H&E analysis of colon tissues (Fig. 1e) showed that DSS-induced colitis caused extended ulcerations, crypt disruption, and transmural inflammatory infiltration, and severely compromising mucosal structure. However, the mice with treatment using ADSC and Rg1 ameliorated colitis-associated damage with the preserved mucosal architecture focal erosions and mild/moderate mucosal inflammatory infiltration. Based on the analysis of the architecture of intestinal mucosa and inflammatory infiltration using histopathology scores to measure of inflammation severity [22], we found that the combined ADSC and Rg1 treatment reduced significantly histopathology scores than that of untreated controls similar to ADSC or Rg1 treatment alone (P < 0.05, Fig. 1f).

### ADSC and Rg1 treatment may ameliorate colitis by regulating pro/anti-inflammatory cytokines

**Fig. 2:**
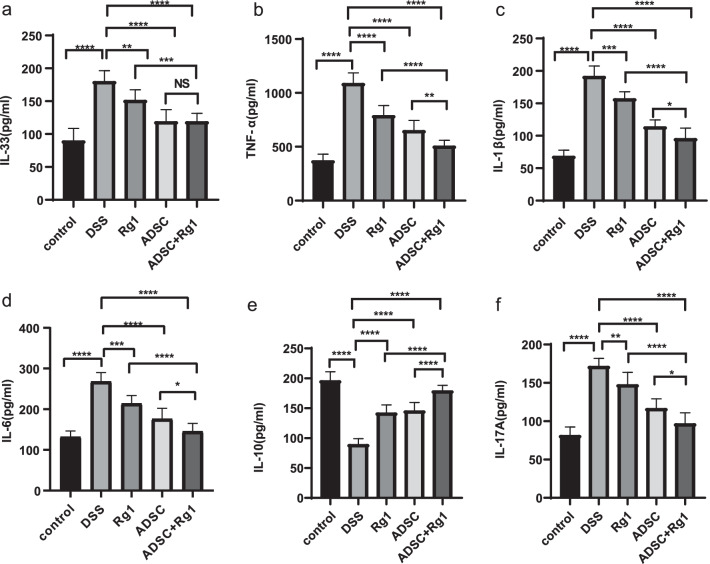
The administrations of ADSC, Rg1, or ADSC + Rg1 alter upregulation of cytokines in DSS-induced colitis mice. Serum cytokines were detected in each group. **a** IL-33 (pg/ml). **b** TNF-α (pg/ml). **c** IL-1β (pg/ml). **d** IL-6 (pg/ml). **e** IL-10 (pg/ml). **f** IL-17A (pg/ml) n = 8, **P < 0.01, ***P < *0.001*

Proinflammatory cytokines play crucial roles in the progression of DSS-induced colitis [26]. To explore if ADSC and Rg1 treatment can ameliorate colitis by regulating inflammation, we compared cytokine levels in sera among the five groups. We observed that the levels of inflammatory cytokines IL-6, IL-33, TNF-α, IL-1β, and IL-17A were significantly lower in Rg1, ADSC, and ADSC + Rg1 groups, while IL-10 levels were elevated compared with DSS group (Fig. 2a-h). Moreover, the group treated with ADSC + Rg1 had significantly lower levels of inflammatory cytokines than the groups treated with Rg1 or ADSC alone (Fig. 2a–h), indicating that Rg1 enhances the effect of ADSC on DSS-induced mouse colitis.

### ADSC and Rg1 regulated Treg/Th17 balance to maintain intestinal homeostasis

**Fig. 3:**
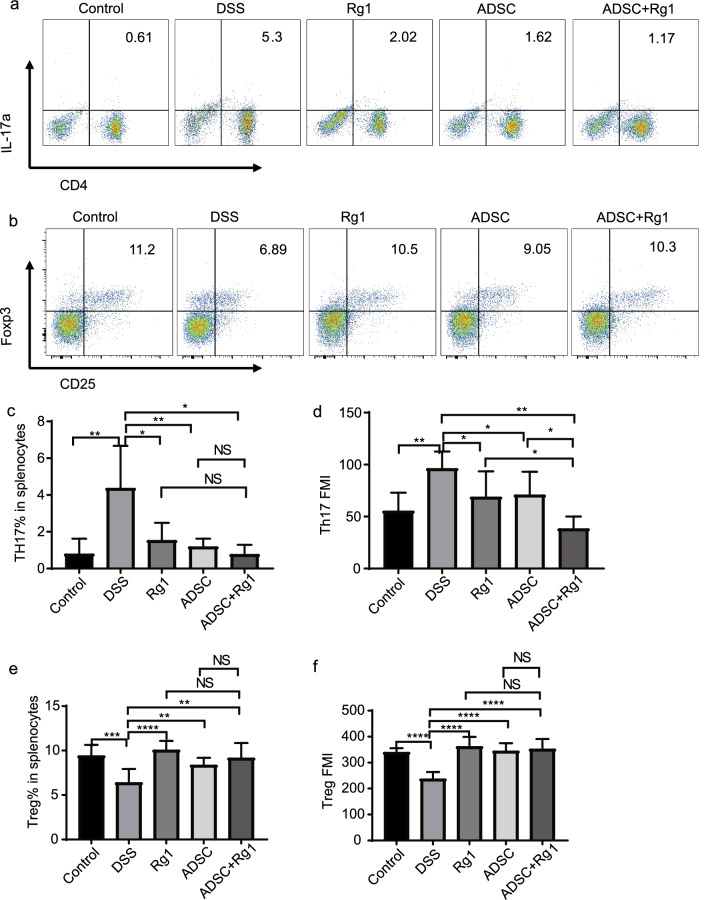
The ADSC and Rg1 improved Treg/Thl7 balance in the spleen in DSS-induced colitis mice through flow cytometry. Cells were surface stained with anti-CD3, anti-CD4, and anti-CD25, and intracellularly stained with anti-IL-17A and anti-FoxP3. (**a**, **b**) Representative dot plots from spleen. (**c**, **e**) The percentage of Th17 cells (CD3^+^CD4^+^ IL-17A^+^) and Treg (CD4^+^CD25^+^Foxp3^+^). (d, f) The MFI of Th17 cells (CD3^+^CD4^+^ IL-17A^+^) and Treg (CD4^+^CD25^+^Foxp3^+^). * *P* < 0.05, **P < 0.01, ***P < *0.001*

Previous studies have shown that ADSCs may inhibit Th17 responses in T cell-mediated autoimmune diseases [27]. ADSCs are also reported to inhibit Treg/Th17 differentiation in a DSS-induced mouse model [15]. Next, we compared the number of Th17 cells between groups and found they were much higher in the DSS group than in the Rg1, ADSC, or ADSC + Rg1 groups (Fig. 3b-c, e). However, the proportion of Treg cells was markedly higher in Rg1, ADSC, and ADSC + Rg1 groups when compared with the DSS group, indicating that Rg1 and ADSC administration selectively upregulated the levels of Tregs and downregulated the proportion of Th17 cells in DSS-induced colitis, and thus improving the Treg/Th17 balance and maintaining intestinal homeostasis (Fig. 3a, d, f). Moreover, simultaneous treatment with ADSC and Rg1 exhibited better recovery of the Treg/Th17 balance when compared with ADSC or Rg1 alone.

### ADSC and Rg1 treatment significantly altered gut microbiota diversity and composition

**Fig. 4:**
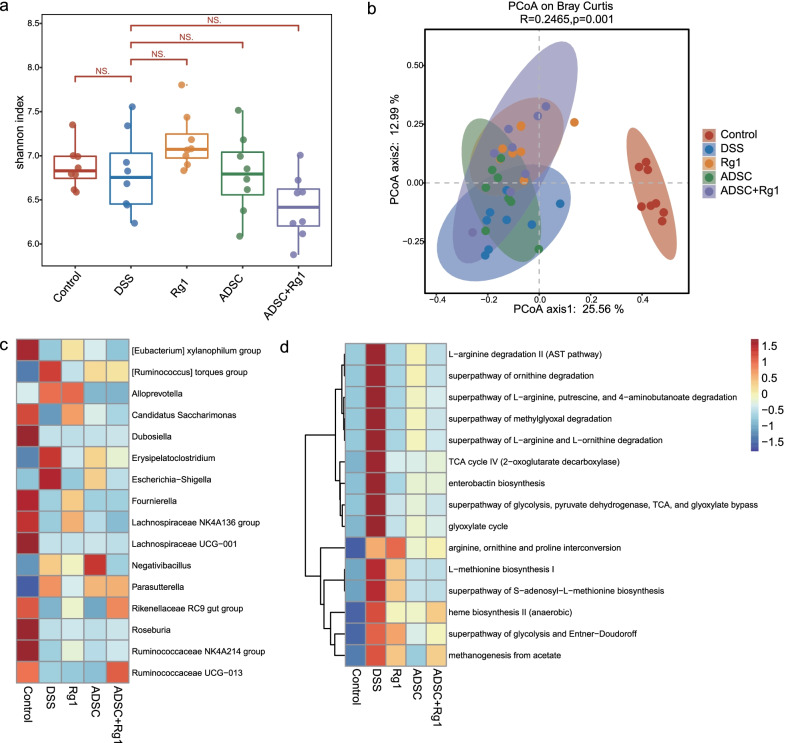
ADSC and Rg1 changed the structure of gut microbiota in DSS-induced colitis mice. **a** Alpha diversity boxplot of the Shannon index in each group. **b** Phylogenetic distances between samples from five groups were figured out by Unweighted UniFrac PCoA (principal coordinates analysis) of the overall gut microbiota. **c** Heatmap of normalized relative abundance levels of OTUs in each group. **d** The average abundance of 15 KEGG modules differentially enriched in each group. **e** Correlation analysis of these differential genera and cytokines between any two groups. n = 8

Gut microbiota is an important regulator of intestinal inflammation [28]; accordingly, we compared microbiota composition among the five groups. Analysis of the alpha diversity of bacterial communities using Shannon’s diversity index revealed that diversity indexes of the ADSC and Rg1 groups did not differ significantly from the DSS group (Fig. 4a,* P* ≥ 0.05) and a PCoA scatterplot revealed clear clustering of gut bacterial communities between the five groups (Fig. 4c). Kruskal–Wallis statistical comparison of gut microbiota composition in the five groups at the genus level revealed that the gut microbiota in the ADSC, Rg1, and ADSC + Rg1 groups was characterized by higher levels of *Rikenellaceae RC9* and *Ruminococcaceae UCG-013* (Fig. 4d) and lower levels of *Erysipelatoclostridium* and *Escherichia-shigella* (Fig. 4d) in comparison with the DSS group. Interestingly, the proportions of *Rikenellaceae RC9*, *Ruminococcaceae UCG-013*, *Erysipelatoclostridium,* and *Escherichia-shigella* in the ADSC + Rg1 group were more similar to the control groups. In addition, we found that the effect of ADSC on gut microbiota disturbance induced by DSS was not significantly improved, and there was even a worsening trend. However, Rg1 restored the disturbed gut microbiota induced by DSS treated by ADSC.

We also observed significant changes in multiple bacteria-associated functional pathways among the five groups. As shown in Fig. 4e, the 15 modules in the five groups were involved in L-arginine degradation, superpathway of L-arginine, putrescine, 4-aminobutanoate degradation, superpathway of methylglyoxal degradation, glyoxylate cycle, citrate cycle (TCA cycle), L-methionine biosynthesis, heme biosynthesis, and methanogenesis from acetate. The overrepresentation of the L-arginine degradation pathway in the DSS group may be due to the elevated abundance of *Escherichia-shigella*, which has been shown to have a high capacity for degrading polysaccharides [29]. Taken together, the microbial function analysis indicated that gut dysbiosis may induce a disease-linked state by interfering with physiological metabolic functions.

### Effects of Rg1/ADSC combination on TLR4-MyD88 signaling

**Fig. 5:**
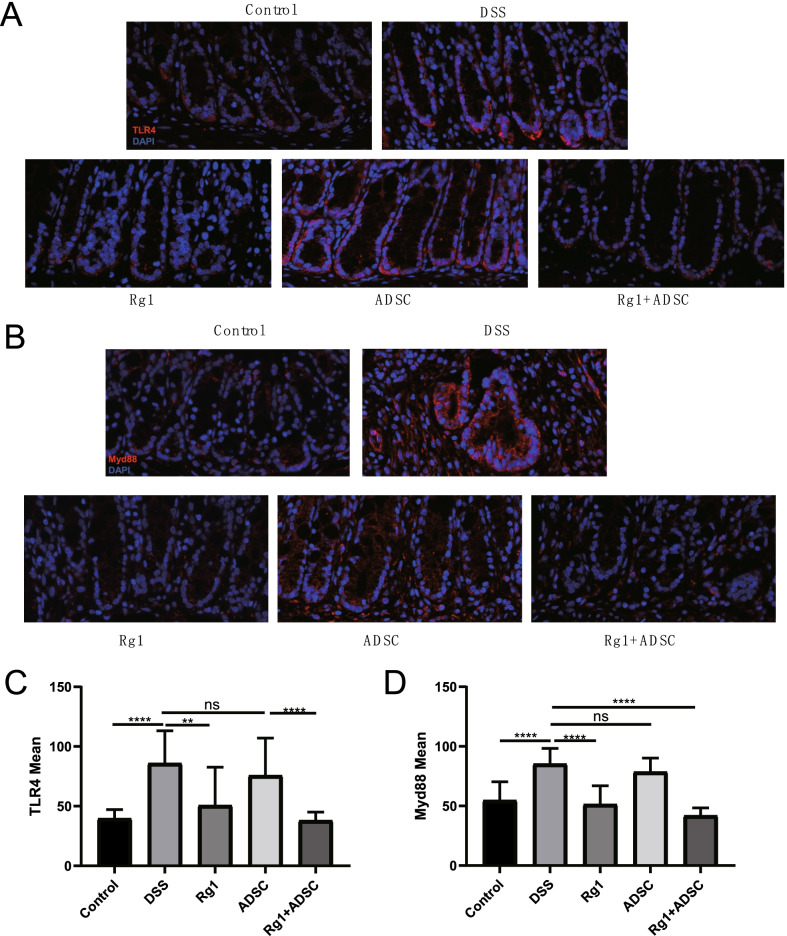
Rg1 and ADSC administration developed the anti-inflammatory activity though the TLR4-MyD88 pathway in DSS-induced colitis. Representative images of the expression of TLR4 and MyD88 in five groups as determined by immunofluorescence. TLR4, MyD88 (red), DAPI (blue), (**a**, **b**). The statistics of fluorescence intensity of immunofluorescence staining in five groups. n = 4 (**c**, **d**). **P < *0.01*, ***P < *0.001*

We found that the combined administration of Rg1 and ADSC affects TLR4-MyD88 signaling. As shown in Fig. 5, the levels of MyD88 and TLR4 were significantly elevated in the DSS-treated group when compared with the control group (all P ≤ 0.01). However, their levels did not differ significantly between the DSS and ADSC groups. Interestingly, pretreatment with Rg1 or ADSC + Rg1 significantly suppressed the expression of TLR4 and MyD88 (all P < 0.01 vs. DSS), indicating that pretreatment with Rg1 and ADSC + Rg1 might downregulate TLR4 and MyD88, thereby suppressing TLR4-MyD8 signaling. These results also suggest that co-therapy Rg-1and ADSC may optimize treatment strategies.

## Discussion

In this study, we found that ADSC/Rg1 coadministration markedly ameliorates colitis when compared to treatment using ADSC or Rg1 alone, as revealed by lower body weight loss, less colon shortening, and better histological scores. Our findings indicate that ADSC/Rg1 coadministration may achieve these effects by: a) repairing the colon mucosal barrier, b) modulating inflammatory response, c) reshaping the gut microbiota, and d) regulating Treg/Th17 differentiation.

Excessive Th17 responses and insufficient Treg function correlate with IBD onset. Previous findings show that ADSCs inhibit Th17 responses in T cell-mediated autoimmune diseases [27] and inhibit Treg/Th17 differentiation in a DSS-induced mouse model of colitis [30]. Consistent with this, we find that ADSC treatment improved the Treg/Th17 balance. Th17 cells produce the proinflammatory cytokine, IL-17A, which contributes to IBD progression [31]. Tregs, which are derived from the thymus, are involved in suppressing innate immune responses [32]. Previous findings showed that improving the Treg/Th17 balance contributes to the re-establishment of intestinal immune homeostasis in IBD [33]. Here, we also found that Rg1 can enhance the effect of ADSCs in improving the Treg/Th17 balance.

Gut microbiota analysis revealed that the microbial community diversity and structure in the ADSC, Rg1, and ADSC + Rg1 groups were significantly changed when compared with the DSS group. For example, in the DSS group relative to other groups, the levels of *Rikenellaceae RC9* and *Ruminococcaceae UCG-013* were reduced, while *Erysipelatoclostridium* and *Escherichia-shigella* were elevated. The *Rikenellaceae_RC9_gut_group* is a dominant group of *Bacteroidetes*, which has been reported to possibly affect intestinal permeability, oxidative stress, energy metabolism, the pathogenesis of acute myocardial ischemia [34], and inflammation response [35]. The *Ruminococcaceae* family is a member of the Firmicutes phylum and comprises a broad spectrum of species with different functional properties. An underrepresentation of species belonging to this family has previously been reported in IBD [36]. For instance, *Erysipelatoclostridium* and *Escherichia-shigella* were reported to play an important role in inflammatory responses [37, 38]. In line with these pieces of previous information, our data show that ADSC transplantation in combination with ginsenoside Rg1 administration significantly improved the ratio of *Rikenellaceae RC*9 and *Ruminococcaceae UCG-013* and reduced the abundance of *Erysipelatoclostridium* and *Escherichia-shigella*, and that its effects were superior to those of ADSC or Rg1 administration alone.

The complex interactions between biological pathways and gut microbiota are intensely associated with host–microbes. Notably, gut microbiota plays an essential role in IBD through various pathways, including glyoxylate cycle, citrate cycle (TCA cycle), and L-arginine degradation pathways [39–41]. Consistent with this, KEGG pathway analysis of the ADSC, Rg1, and ADSC + Rg1 groups was different from DSS groups, such as glyoxylate cycle, citrate cycle (TCA cycle), and L-arginine pathway reduced significantly, indicating that ADSC and Rg1 may modulate these bacteria-associated pathway by restoring the composition of gut microbiota.

Emerging evidence suggested that the inflammatory response was primarily regulated by the TLR4-MyD88 pathway [42]. TLR4, a member of the Toll-like receptor family, is upregulated in animals and humans with IBD [43, 44]. TLR4 activation initiates MyD88 activation, an important adaptor molecule essential for TLR signaling. This in turn triggers the production of downstream proinflammatory cytokines, including IL-6, IL-1β, and TNF-α, which contribute to IBD development [45]. Our data suggest that pretreatment with Rg1 and ADSC + Rg1 markedly decreases the levels of TLR4 and MyD88, as well as the expression of TNF-α, IL-6, IL-1β, IFN-γ, and IL-17A in the DSS-induced mouse model of colitis. Additionally, previous studies have shown that the TLR4-MyD88 pathway highly correlates with gut dysbiosis [46, 47]. Our findings show that ADSC/Rg1 coadministration significantly influences TLR4-MyD88 signaling and the composition to gut microbiota. These results suggest that the beneficial effects of Rg1 and ADSC + Rg1 against DSS-induced colitis may be associated with the improvement of the inflammatory and microbiota status via the regulation of the TLR4-MyD88 signaling pathway.

Taken together, our data suggest that ADSC/Rg1 coadministration may improve IBD by partially restoring the Treg/Th17 balance and the gut microecological structure.

## Conclusion

Overall, our data reveal that Rg1/ADSC coadministration alleviates DSS-induced colitis more efficiently than Rg1 or ADSC alone. The effects may be achieved by restoring the balance of pro/anti-inflammatory cytokines, Treg/Th17 balance, and gut microecological system.

## Supplementary Information

**Additional file 1** **Figure S1**: Phenotypic characterization of ADSCs. (a) Cell phenotype of ADSC. (b) Tri-lineage differentiation of passage 3 ADSC, as described in “Materials and Methods” section.

## Data Availability

The raw sequence data reported in this paper have been deposited in the Genome Sequence Archive (GSA: CRA007023) that are publicly accessible at https://bigd.big.ac.cn/gsa/browse/CRA007023.

## Abbreviations

IBD: Inflammatory bowel diseases
MSCs: Mesenchymal stem cells
ADSC: Adipose-derived stem cell
