# Supplementary figures and images for: Combined treatment with Rg1 and adipose-derived stem cells alleviates DSS-induced colitis in a mouse model
Source: Stem Cell Res Ther. 2022 Jun 21;13:272. doi: 10.1186/s13287-022-02940-x (PMC9210677; doi:10.1186/s13287-022-02940-x)

**a**

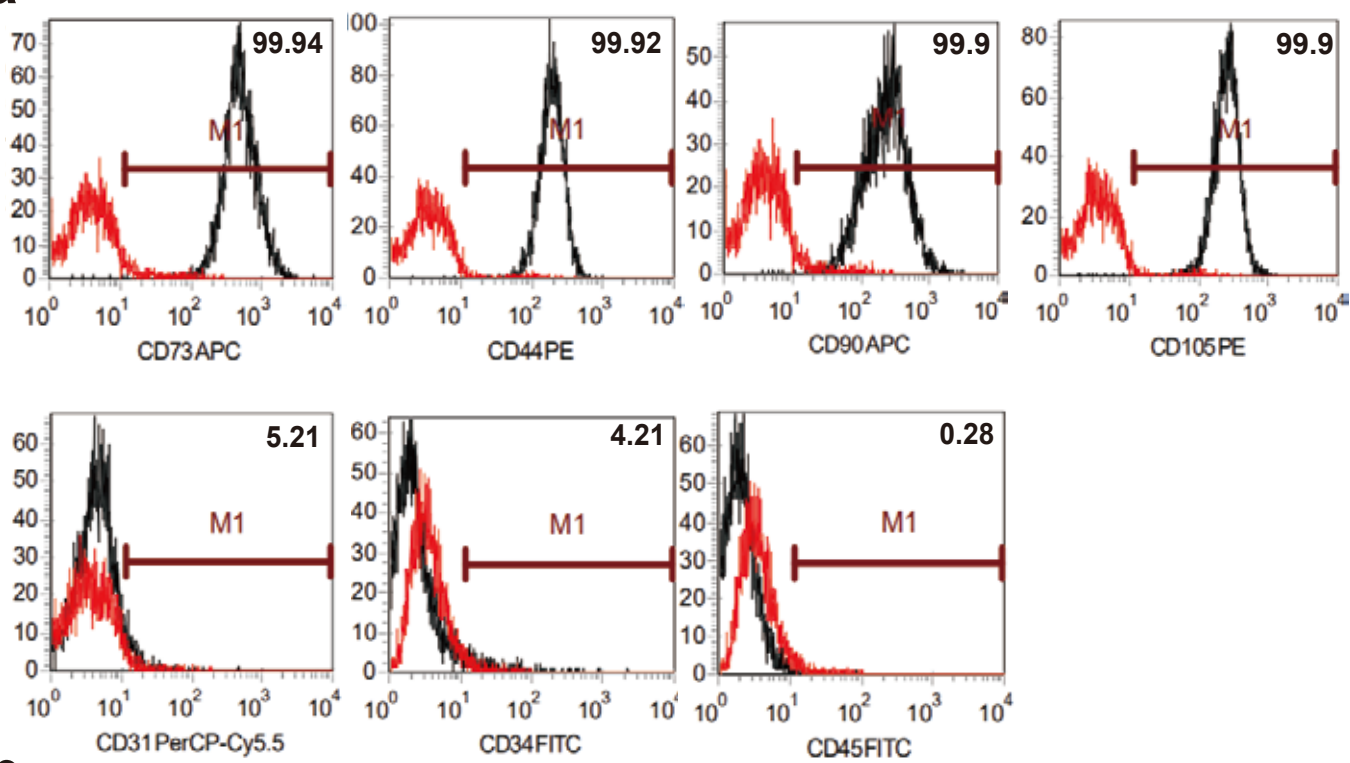

**b**

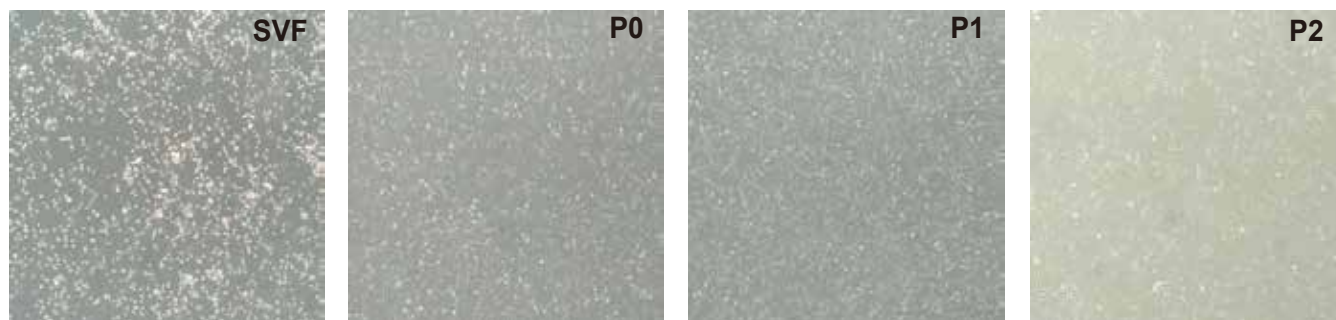

Supplement: Supplementary file 1 — Additional file 1 Figure S1: Phenotypic characterization of ADSCs. (a) Cell phenotype of ADSC. (b) Tri-lineage differentiation of passage 3 ADSC, as described in “Materials and Methods” section. [file 13287_2022_2940_MOESM1_ESM.pdf]
